# Sexually Dimorphic Transcriptomic Changes of Developing Fetal Brain Reveal Signaling Pathways and Marker Genes of Brain Cells in Domestic Pigs

**DOI:** 10.3390/cells10092439

**Published:** 2021-09-16

**Authors:** Monica Strawn, Joao G. N. Moraes, Timothy J. Safranski, Susanta K. Behura

**Affiliations:** 1 Division of Animal Sciences, University of Missouri, Columbia, MO 65211, USA; mpsrkf@mail.missouri.edu (M.S.); SafranskiT@missouri.edu (T.J.S.); moraesjgn@okstate.edu (J.G.N.M.); 2 Department of Animal and Food Sciences, Oklahoma State University, Stillwater, OK 74075, USA; 3 MU Institute for Data Science and Informatics, University of Missouri, Columbia, MO 65211, USA

**Keywords:** fetal sex, gene regulation, brain development, placenta, swine

## Abstract

In this study, transcriptomic changes of the developing brain of pig fetuses of both sexes were investigated on gestation days (GD) 45, 60 and 90. Pig fetal brain grows rapidly around GD60. Consequently, gene expression of the fetal brain was distinctly different on GD90 compared to that of GD45 and GD60. In addition, varying numbers of differentially expressed genes (DEGs) were identified in the male brain compared to the female brain during development. The sex of adjacent fetuses also influenced gene expression of the fetal brain. Extensive changes in gene expression at the exon-level were observed during brain development. Pathway enrichment analysis showed that the ionotropic glutamate receptor pathway and *p53* pathway were enriched in the female brain, whereas specific receptor-mediated signaling pathways were enriched in the male brain. Marker genes of neurons and astrocytes were significantly differentially expressed between male and female brains during development. Furthermore, comparative analysis of gene expression patterns between fetal brain and placenta suggested that genes related to ion transportation may play a key role in the regulation of the brain-placental axis in pig. Collectively, the study suggests potential application of pig models to better understand influence of fetal sex on brain development.

## 1. Introduction

The development of the fetal brain is regulated by complex and highly coordinated spatiotemporal processes [1,2,3]. In pigs, the fetal brain develops in an accelerated manner after gestation day (GD) 60 [4]. Distinct regions of the brain are formed during rapid growth of the fetal brain [5]. The use of pigs as a large animal model for biomedical research is increasing [6,7,8]. In particular, pig models have shown promising utilities in research relating to brain structure and function, including a better understating of neurodegenerative diseases in humans [9,10,11,12]. 

While gene expression is tightly regulated in the brain during development, the sex of the fetus plays an influential role in orchestrating brain development [13,14]. Developmental differences in brain size, head circumference, rate of energy consumption of the brain, and epigenetic modification of brain DNA have been reported in humans [15,16,17,18]. The early organization of the vertebrate brain occurs in a sex-dependent manner, and is under the control of the perinatal gonadal steroid hormones [19,20,21,22]. Gene expression has been widely applied to investigate brain development in animals [2,23,24,25] and to identify the role of sex during brain development [14]. RNA-sequencing (RNA-Seq) has been particularly useful to identify the presence and prevalence of gene splice forms in the brain [26,27].

Recent studies further suggest that brain development is reliant on placental function [28]. In humans, placental dysfunction can cause defective neuronal development that increases risk of neuropsychiatric diseases later in life [29]. In mice, our earlier study showed that there is a remarkable coordination in the gene expression between the placenta and fetal brain [30]. The brain–placental axis plays important roles in the fetal programming of brain development [31]. Placentotrophy is considered as the most wide-spread form of matrotrophy in viviparous animals [32,33]. However, there are striking differences in the feto–maternal interface among placental animals [34]. For instance, the pig placenta is diffused as placentation occurs all over the allantochorion. It is also epitheliochorial [35], as there are different tissue layers that separate direct contact of the fetus from the maternal blood. On the other hand, the mouse placenta is discoid and hemochorial (same as humans) [36]. 

While research using mice models has provided a wealth of information into the processes of brain development [3], the use of large animal models to study fetal brain development is lacking. With an increase in use of pigs as a large animal model for brain research [11], the identification of genes that are differently regulated during pig brain development in male and female fetuses is of significant research interest. Earlier study by Dickerson and Dobbing (1967) [4] showed that a pig’s fetal brain grows rapidly ~50 days before birth, which is around GD 60. So, we wanted to investigate changes in gene expression of the male and female fetal brain on GDs 45 (before rapid growth), 60 (during rapid growth) and 90 (after rapid growth). The specific objectives of the current study are to (1) identify marker genes of brain cells and signaling pathways associated with the onset of rapid brain development in pig fetuses, (2) determine the influence of fetal sex on gene expression of the developing brain and (3) identify genes coordinately expressed between the pig placenta and fetal brain.

## 2. Materials and Methods

### 2.1. Animals and Fetal Brain Collection

Gilts (Landrace × Large White) were obtained from the University of Missouri Swine Research Teaching and Research Farm and bred to LR-M6 from Choice Genetics (West Des Moines, IA, USA) by artificial insemination (AI). GD 1 was considered the day of AI. Dams were euthanized on GD45, GD60 or GD90 via electrical stunning and exsanguination by the University of Missouri abattoir, an USDA inspected commercial unit (Establishment #5077A). The reproductive tract was removed from the pregnant dam at the abattoir and transported to the lab for dissection of fetuses. Individual fetuses of both sexes were removed from each uterine horn. No fetuses were taken from the tip of either horn or the body of the uterus. While collecting fetuses, the sex of the fetus to be included in gene expression analysis was recorded along with the sex of the fetus on the medial side (closer to the body of the uterus) and also the sex of the fetus on the lateral side (closer to the tip of the uterus horn). As there was no information on which specific brain regions were important for sex differences during brain development in pig, the whole brain from each fetus was dissected in this study. The weight of the fetus as well as the dissected brain was recorded. In total, 18 fetal brain samples (3 gestation days × 2 sexes × 3 biological replicates) were collected to perform transcriptomic analysis. 

### 2.2. Transcriptome Profiling by RNA-Seq 

Each fetal brain was homogenized with RLT buffer (QIAGEN, Germantown, MD, USA) supplemented with 200 μL 2-mercaptoethanol. The RLT buffer amount was scaled up by 1 mL per each mg of brain weight across all samples, and the brain tissue was homogenized using a benchtop VDI 25 tissue homogenizer (VWR, Radnor, PA). Total RNA was isolated using the QIAGEN AllPrep DNA/RNA Mini Kit as per manufacturer’s instruction. Briefly, the homogenate was centrifuged for 1 min and the clear lysate (750 μL) was transferred to RNase/DNase-free 1.7 mL conical tubes (Corning, Tewsksbury, MA, USA) and mixed with 350 μL 70% ethanol to precipitate RNA. DNase treatment was performed to clean up genomic DNA contaminants. RNA was eluded in 30 μL nuclease-free water twice for a total volume of 60 μL. Concentration and purity of RNA was determined using a Nanodrop 1000 spectrophotometer (Thermo Fisher Scientific, Waltham, MA, USA), and then each sample was diluted to 100 ng/μL using nuclease-free water. RNA integrity was measured by Agilent 2100 (Agilent Technologies, Santa Clara, CA, USA). Total RNA of each sample was processed to prepare Illumina sequencing libraries by the Novogen Cooperation Inc. (Sacramento, CA, USA). Each library was sequenced to a depth of 20 million paired end reads of 150 bases using NovaSeq 6000. 

### 2.3. RNA-Seq Data Analysis

The quality of raw sequences was checked with *FastQC* tool (v0.11.9, Babraham Institute, Cambridge, UK). The sequences were subjected to quality control using the Trimmomatic tool (v0.39) as described earlier [37]. The reads were then mapped to the pig reference genome Sscrofa11.1 using *Hisat2* (v 2.2.0) aligner [38]. The number of reads that mapped to the annotated genes (Ensembl annotation, Sus_scrofa.Sscrofa11.1.104.gtf) in each sample was determined from the sequence alignments by using the *FeatureCounts* tool (v1.5.0-p1) [39]. The raw and processed data of the RNA-Seq are publicly available in the GEO database (accession # GSE178970). The count data were subjected to paired-sample differential gene expression analysis by *edgeR* (v3.28.0) [40] to identify the differentially expressed genes (DEGs).

### 2.4. Functional Annotation of Differentially Expressed Genes

Gene ontology and pathway enrichment analysis was performed by a Fisher exact test followed by multiple correction of raw *p*-values of significance to false discovery rates (FDR) using the PANTHER Classification System (v16.0) [41]. 

### 2.5. Analysis of Marker Genes of Brain Cells

The marker genes predicted from mammalian brain single-cell RNA-Seq projects, available in the PanglaoDB [42], were used to annotate cell types of differentially expressed (DE) marker genes. The marker genes were downloaded from the database (https://panglaodb.se/, accessed on 28 January 2020), and compared with the DEGs identified from the current study. To further compare genes differentially expressed in the brain of pigs with human data, the developmental transcriptome data of the human fetal brain (*RNA-Seq Gencode v10 summarized to genes*) was downloaded from the human BrainSpan database (https://www.brainspan.org/static/download.html, accessed on 8 September 2021). Significant differences in gene expression of the developing human fetal brains of males and females (age-matched: 13- and 21-weeks post-conception) were determined from that data by edgeR. The orthology of the identified significant (*p* < 0.05) genes of humans relative to pigs was determined from Ensembl homology annotation via BioMart. The purpose of this analysis was to know if the same gene was significantly differentially expressed in the developing brain between male and female fetuses of humans and pigs.

### 2.6. Statistical Analyses 

The hierarchical cluster analysis of gene expression was performed using the R package dendextend (v1.13.2) [43]. To infer gene expression networks within a predicted cluster, the mutual information (MI) analysis [44] was performed. In this method, MI values of gene expression variation were calculated in a pair-wise manner to infer expression networks using R package minet (v3.44.0) [45]. Canonical correlation analysis of gene clusters between males and females was performed using the CCA package (v1.2.1). All statistical analyses and plotting were performed using base functions in R (v3.6.2).

## 3. Results

### 3.1. Developmental Changes in Gene Expression of Fetal Brain

**Figure 1:**
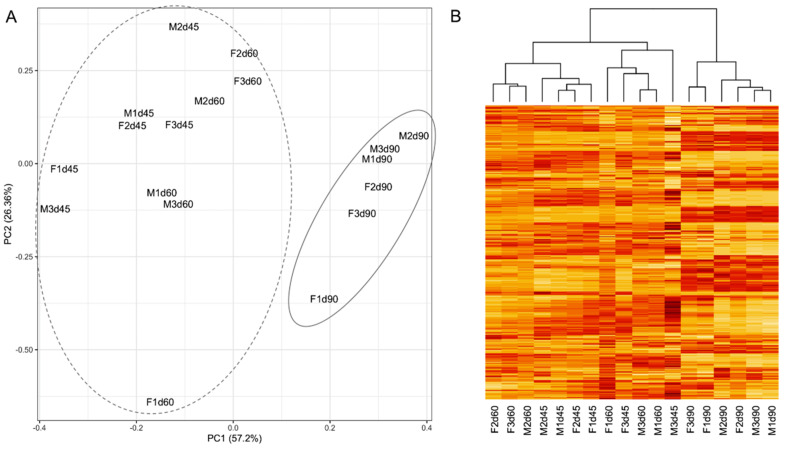
(**A**) Principal component analysis of gene expression variation of male (M) and female (F) fetal brains on GDs 45, 60 and 90. The dotted ellipse shows the group representing gene expression of fetal brain on GDs 45 and 60, and the solid ellipse shows the group representing gene expression of fetal brain on GD 90. (**B**) Heatmap of expression of genes in fetal brain samples also shows that GD 90 samples cluster differently than the samples of GDs 45 and 60.

**Figure 2:**
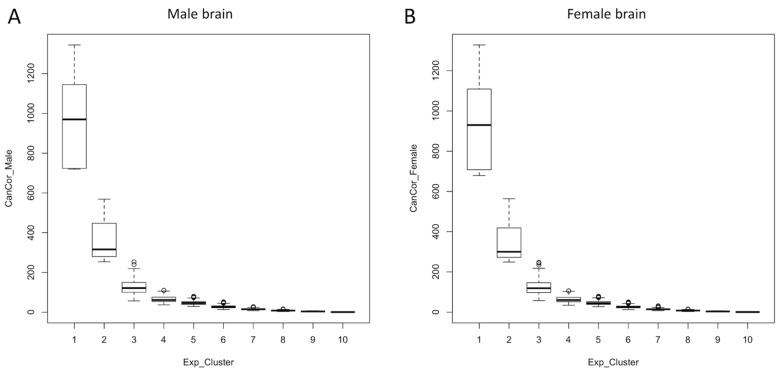
Box plots of covariates showing canonically correlated expression clusters between male brain (**A**) and female brain (**B**). The x-axis shows expression clusters (1–10) and y-axis shows covariates of canonical correlation. The mean value of expression cluster is shown by horizontal line inside each box. The lower and upper quartile values of covariates are shown for each cluster.

Gene expression of fetal brain samples was profiled on GDs 45, 60 and 90. Principal component analysis (PCA) showed that GD90 fetal brains were transcriptionally distinct from that of GDs 45 and 60 (Figure 1A). Nearly 57% of the variation in gene expression was explained by the first principal component. Heat map of global expression changes, shown in Figure 1B, also supported the differential gene expression in GD90 samples compared to GDs 45 and 60 which is shown in the hierarchical cluster [43] relationship among the brain samples. In addition, canonical correlation analysis (CCA) [46] was performed between male and female brains to compare gene expression clusters (Figure 2). This analysis showed that gene expression changes in a pig’s fetal brain are regulated in modular patterns in both sexes. This finding is consistent with results of previous studies that also observed modular expression patterns of the developing brain in other mammals, including humans and mice [47,48].

### 3.2. Influence of Fetal Sex on Gene Expression Changes of Brain

**Figure 3:**
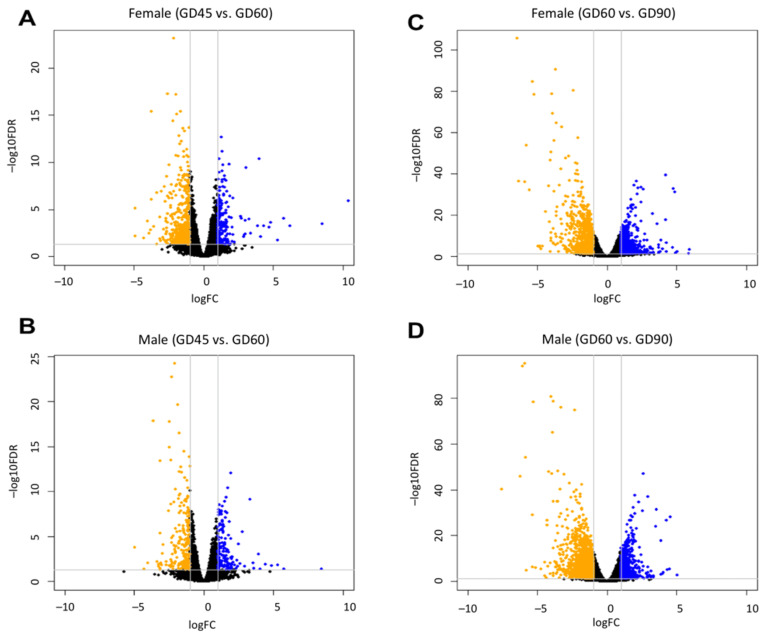
Volcano plots showing differential expression of genes in female fetal brain between GDs 45 vs. 60 (**A**), male fetal brain between GDs 45 vs. 60 (**B**), female fetal brain between GDs 60 vs. 90 (**C**), and male fetal brain between GDs 60 vs. 90 (**D**). In each plot, the y-axis shows the –log10(FDR) values and x-axis shows log fold change values. The orange color shows genes that are downregulated, and blue color shows genes that are upregulated between the two groups in each plot. The horizontal line above value 0 in y-axis represents FDR value of 0.05 used to identify significance of differential expression of genes.

The volcano plots in Figure 3 show the number of DEGs in the fetal brain of males and females during development. Greater number of DEGs was observed in the brain during development from GD60 to GD90 compared to that from GD45 to GD60, and this bias was reliant on the sex of the fetuses (Table 1). The data in Table 1 shows a significant bias (Chi-square = 278.5, *p* < 0.0001) in the number of genes that are differentially expressed in the male brain compared to the female brain during development. The list of these DEGs is provided in Table S1. Of the 2059 and 1323 genes that altered in the female and male brain respectively during GD45 to GD60, 785 genes were commonly DE in the brain of both sexes. Similarly, of the 5325 and 6605 genes that altered in the female and male brain respectively during GD60 to GD90, 2767 genes were commonly DE in the brain of both sexes. There were 529 genes that altered in the brain of both males and females at both the developmental periods (GD45 to GD60 as well as GD60 to GD90). These common DEGs are indicated in Table S1. We also identified genes that showed a significant difference (FDR < 0.05) between male and female brains at each GD (Table S2). Some of these genes were DE between males and females at GD45 as well as GD60. A total of 11 genes were commonly DEGs between males and females across the three GDs. We further wanted to know how the genes differentially expressed in the pig fetal brain are expressed in the human fetal brain. To explore that, the developmental transcriptome data of the human fetal brain (*RNA-Seq Gencode v10 summarized to genes*), obtained from the human BrainSpan database (https://www.brainspan.org/static/download.html), was analyzed in edgeR to identify significant differences in the gene expression of the developing human fetal brain of males and females (age-matched: 13 weeks and 21 weeks post-conception). Then, orthology analysis of the identified significant (*p* < 0.05) genes relative to pig genes was performed, which found genes (n = 40) that were commonly DE between the male and female fetal brains of both humans and pigs. Though these genes were commonly differentially expressed, the direction was not always conserved between the two species (Table S3). Based on Ensembl prediction of the last common ancestry of those genes, the majority of those 40 genes have the last common ancestors in Boreoeutheria magnorder of placental animals. Five of those common DEGs (shown in Table S3) are associated with Wnt signaling, which plays crucial roles in brain development [49].

### 3.3. Effect of Adjacent Fetus on Gene Expression of Brain

In litter-bearing mammals, the sex of neighboring fetuses in the uterus influences fetal development [50]. A female fetus developing between two males shows masculinized anatomical and physiological traits whereas a female fetus developing in the absence of adjacent males, tends to show feminized traits [51]. To evaluate whether the sex of adjacent fetuses influenced developing fetal brain transcriptome, we made use of recorded data on the sex of adjacent fetuses of the medial side and the lateral side of the uterus during sample collection. In our collection, we identified male fetuses (n = 3) that were flanked by two female fetuses (fMf). We also found male fetuses (n = 3) of the same gestational ages (GDs 45 and 60) where there was a male on the medial and a female on the lateral side (mMf). Thus, we wanted to know if the brain of those male fetuses with differential adjacent fetuses (fMf vs. mMf) had differential gene expression. The differential gene expression analysis by *edgeR* showed a total of 54 genes (Table S4) that were impacted in the fetal brain of the males when they were flanked by fetuses of both sexes (mMf) as opposed to fetuses of same sex (fMf). Several of these genes (37 out of 54 genes) showed significantly (FDR < 0.05) higher expression in the brain of the fMf males relative to the mMf males. Though none of these genes were related to any sex hormone, we identified that 16 of these genes were ion transporters (see Table S4), a class of transporters that play important roles in feto–placental communication [52,53]. 

### 3.4. Bias in Exon-Level Expression of Fetal Brain

Analysis of exon-level expression identified genes in which one or more exons were significantly differentially expressed during fetal brain development (Table S5). The counts of upregulated and downregulated exons during brain development are shown in Table 2. The data further showed that the second and third exons in those genes were more likely to be differentially expressed than other exons, irrespective of the fetal sex. Exon-rich genes showed an inverse relationship between expression level and the rank order of exons within the genes. The higher ranked exons were less likely to be differentially expressed in the fetal brain (Table S5), suggesting that the number of exons within a gene is a potential predictor of expression of that gene in the fetal brain. In addition, exons within the same gene showed differential expression patterns during brain development (Table S6), which indicated that these exons are more likely to influence the protein isoforms encoded by these genes. 

### 3.5. Functional Annotation of Differentially Expressed Genes

Gene ontology (GO) analysis showed significant enrichment of different biological functions that were associated with significant enrichment (Exact test, *p* < 0.05) among the genes differentially expressed in the fetal brain between GD40 vs. GD60 and GD60 vs. GD90 (Table S7). The data in Table S7 shows the specific GO terms commonly enriched in the brain of both sexes. It also shows GO terms enriched exclusively in either the female brain or male brain. Functions related to cerebral cortex regionalization, pre-replicative complex assembly involved in cell cycle DNA replication, pre-replicative complex assembly involved in nuclear cell cycle DNA replication, and double-strand break repair via break-induced replication were among the top 10 significant GO terms in the fetal brain of both sexes during development from GD45 to GD60. However, as the brain developed from GD60 to GD90, functions related to glutamate secretion, regulation of axon extension involved in axon guidance and regulation of neuronal synaptic plasticity were found as the top 10 significant GO terms. We found several enriched GO terms (n = 41) that were related to ion transport (Table S7). Ion transporter genes play a role in feto–placental communication and circulation, possibly by regulating the placental–blood barrier [54,55,56,57,58]. So, we were interested to know if the expression of these genes were regulated between the fetal brain and placenta. Towards that objective, the gene expression data of the fetal brain on GD60 and GD90 from the current study were compared with transcriptomic data (accession number GSE110414) generated at the same gestation days from pig placentae in a previous study [32]. The comparative analysis identified genes (n = 1275) that were expressed either in the placenta or fetal brain in an ‘ON/OFF’ manner (Table S8). We identified 45 of these genes expressed either in the placenta or fetal brain that were related to ion transporters (shown in Table S8), further suggesting that ion transporter genes were tightly regulated in the placenta relative to the fetus during brain development.

In addition to GO analysis, we also performed PANTHER pathway enrichment analysis to identify if specific pathway(s) were over-represented by DEGs in the fetal brain (Table 3). This analysis showed that endothelin signaling, gonadotropin-releasing hormone receptor signaling, angiogenesis and Wnt signaling were commonly associated with pig brain development. The ionotropic glutamate receptor pathway and *p53* pathway were enriched in the female brain but not in the male brain. On the other hand, different receptor-mediated signaling pathways were enriched in the male brain but not in the female brain (Table 3). The relevance of sex-biased enrichment of signaling pathways in brain development is further described in the discussion section.

### 3.6. Identification DE Marker Genes of Brain Cells

**Figure 4:**
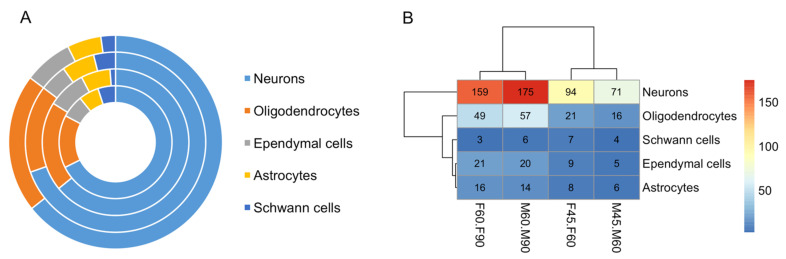
(**A**) Donut plot showing proportion of marker genes representing different brain cells (color coded). The four circles in this donut plot represent the samples in which the marker genes were significantly differentially expressed. From the center to outward direction, these circles represent DE genes between brain samples of F45 vs. F60, F60 vs. F90, M45 vs. M60, and M60 vs. M90, respectively. F and M represent female and male respectively whereas the numbers represent GDs. (**B**) Heatmap showing the number marker genes associated with different cell types of the brain (shown in rows) and GDs during which those genes are significantly differentially expressed in the male and female fetal brain (shown in columns). The cluster patterns of rows and columns are shown along with color scale (in the right). The scale represents the number of DE marker genes of brain cells.

**Figure 5:**
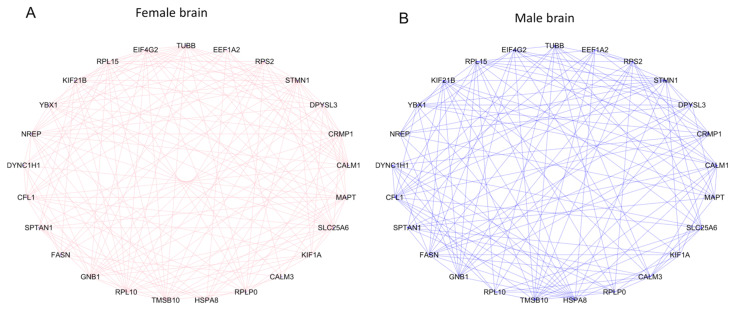
Network plot of genes based on mutation information of expression changes during development of female fetal brain (**A**) and male fetal brain (**B**). The lines (edges) connecting the nodes show how a gene is transcriptionally interconnected with other genes. They are colored pink in female brain and blue in male brain.

**Figure 6:**
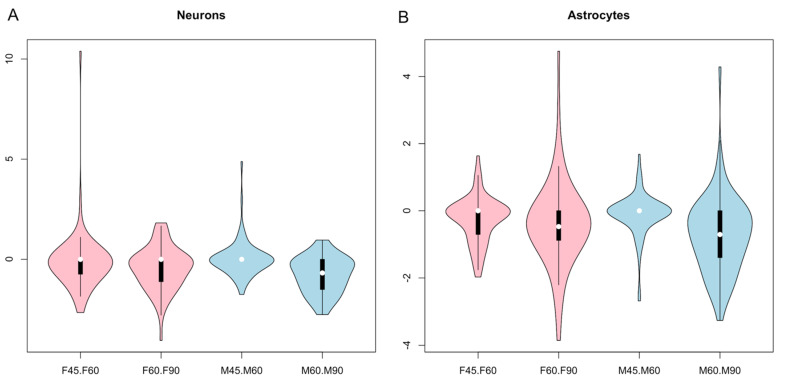
Violin plots of e×pression of marker genes specific to neurons (**A**) and astrocytes (**B**) of male (light blue) and female brain (pink) developing during GD45 to GD60 and GD60 to GD90. In both plots, the values in y-a×is represent the log fold changes of gene e×pression. The ×-a×is shows the sample comparisons.

We performed gene set enrichment analysis of DE marker genes to predict cell types of the brain. The known marker genes of different brain cells were obtained from the PanglaoDB [42], and those showing 1:1 orthology to the differentially expressed genes identified from our current study were used to predict cell types. This analysis identified 357 marker genes of neurons, oligodendrocytes, ependymal cells, astrocytes and Schwann cells that were differentially expressed during the development of both male and female brains (Table S9). Markers of neurons and oligodendrocytes were relatively more abundant than markers of other cell types (Figure 4A). The marker genes that altered between GD60 and GD90 were relatively more abundant than those altered between GD45 and GD60 (Figure 4B). We identified specific neuronal maker genes that were expressed in a se×-biased manner (Table S9). Expression network analysis [45] further showed that these marker genes were expressed in a mutually informative manner [44] in the male and female fetal brain (Figure 5). The marker genes (n = 25) shown in Figure 5 vary in a mutually informative manner among each other in the fetal brain of both sexes. A comparison of the expression level of marker genes of neurons and astrocytes (Figure 6) showed that these marker genes were expressed differentially between male and female fetal brains during development.

## 4. Discussion

It is known that the rate of growth of male and female fetuses vary throughout gestation [59]. Birth weight is generally higher in male than female piglets [60]. Sex-mediated differences in growth is well documented in other animals including humans [61,62,63,64]. Our current study showed fewer numbers of DEGs in the fetal brain during GD45 vs. GD60 compared to GD60 vs. GD90 developmental period. There was a ~3.5-fold increase in the number of genes that altered between GD60 and GD90 compared to GD45 and GD60 (Table 1). This finding implied that gene expression changed extensively in the fetal brain after GD60 when the brain starts growing rapidly [4]. This is consistent with observation from earlier study [65]. The greater number of DEGs in late gestation is suggestive of preparation of the brain for postnatal function [66]. The homeobox *HOXA5* gene that regulates pattern formation in early development [67] was one of the top upregulated DEGs between GD45 to GD60 in the fetuses of both sexes. However, *HOXB5* and *HOXD3* genes were among the top upregulated genes between GD45 and GD60 of the fetal brain of females, but not males. The myelin associated glycoprotein was among the most downregulated DEGs in both male and female brains during GD60 to GD90 development. Myelin associated glycoprotein maintains the myelin–axon spacing by interacting with specific neuronal glycolipids, inhibiting axon regeneration, and controlling myelin formation [68]. The gene coding for *ERMN* that plays a role in cytoskeletal rearrangements, and brain myelination in humans and mice [69] was one of the top downregulated DEGs between GD60 and GD90 in both male and female fetuses.

In pigs, the relationship between intrauterine position of the fetus and fetal development was implied from an earlier study [70]. A subsequent study [71] found that fetuses on each end of the uterine horn grow faster than the fetuses on the medial site of the uterus, but suggested that sex of the adjacent fetuses has lesser influence than the absolute intrauterine position on the fetal growth in pigs. Given this dichotomy about fetal development and fetal location in the uterus, we wanted to determine if sex of the adjacent fetuses had an influence on gene expression of the fetal brain. During sample collection, we identified male fetuses that were either flanked by two females or by a male on medial side and a female on the lateral side. Gene expression of the developing brain was significantly altered in male fetuses flanked by two females compared to fetuses flanked by a male on one side and a female on the other side. As many as 54 genes were impacted, suggesting that sex of the adjacent fetuses had an influence on brain development. 

Pathway enrichment analysis identified specific signaling pathways that were significantly over-represented among DEGs in the fetal brain (Table 3). This analysis showed that the *p53* pathway was enriched in the female brain only. Multiple studies using mice models have shown that *p53* plays a seminal role in protecting brain development in females only [72,73,74]. In particular, it was found that a loss of *p53* can cause neural tube defects in female embryos, likely due to the dysregulation of × inactivation [73,75]. In addition to *p53*, the ionotropic glutamate receptor pathway was also found significant in the female brain only. The ionotropic glutamate receptor system (that includes the subtypes of a-amino-3-hydroxy-5-methyl-4-isoxazolepropionic acid receptors, N-methyl-D-aspartate receptors and kainate receptors) are known to play important roles during brain development as defects of these receptors induce diverse neuronal disorders in a sex-specific manner [76]. Studies using rat models have also shown that ionotropic glutamate receptor pathway regulation, particularly mediated by N-methyl-D-aspartate receptors, induces female specific developmental defects in brain [77,78,79]. We identified histamine H1 receptor mediated signaling pathway, metabotropic glutamate receptor group III pathway, heterotrimeric G-protein signaling pathway-Gq alpha and Go alpha mediated pathway, heterotrimeric G-protein signaling pathway-Gi alpha and Gs alpha mediated pathway, EGF receptor signaling pathway and CCKR signaling map that were enriched among DEGs of the male fetal brain only (Table 3). Histamine plays key regulatory roles in controlling specific cell types of brain that express histamine G protein-coupled receptors [80]. Moreover, histamines mediate pro- as well as anti-inflammatory responses to cytokines. Studies have shown that activation of the maternal immune response can alter neurodevelopmental processes in a sex-biased manner that influences brain development only in males [81]. The cholecystokinin (*CCK*) is abundantly expressed in the brain, and the G-coupled protein receptors of *CCK* play crucial synergetic signaling functions during brain development [82]. Study [83] also suggests that the epidermal growth factor (EGF) receptor signaling has sex-biased influence on brain development. These literature evidences support the sex-biased enrichment of signaling pathways we identified in our current investigation (Table 3). More importantly, our data showed that specific pathways, such as endothelin signaling pathway, gonadotropin-releasing hormone receptor pathway, angiogenesis and *Wnt* signaling pathway, play common roles in the brain development of both sexes, which is also supported from results of earlier studies [83,84,85,86]

Our study was limited in the scope. A major limitation of this study was the small sample size (18 only) for which we couldn’t identify different combinations of adjacent fetal sexes to investigate their effects on brain development. Furthermore, differences in the brain of males versus females could be due to physiological factors during gestation, likely related to neuroendocrine- or sex-hormones [87,88]. At the same time, evolutionary developmental (Evo-Devo) factors can also influence the intrinsic regulation mechanisms of brain development in a species [89]. If specific processes of the central nervous system develop faster in one sex over the other, though this remains unknown, is also an important question relevant that has relevancy to aging of the brain. It is known that developmental differences in early life can influence the aging process of the brain later in life [90]. In humans, it is known that the brain of women remain metabolically more younger than the brain of men during aging [91]. If this is a consequence of sex differences in brain development at the fetal stages remains unknown. Additional studies are required to address these relevant questions regarding fetal brain development. 

Nevertheless, our study revealed an important finding about the association of ion transporter genes with fetal brain development. A comparative analysis of gene expression data between GD60 and GD90 identified 45 ion transporter genes that were coordinately regulated in the form of ON/OFF expression between the fetal brain and placenta. Ion transporters play crucial roles in fetal development [52,53,56,58]. They not only control the placental–blood barrier but also modulate the feto–placental circulation and molecular communication [53,56]. 

## 5. Conclusions

In conclusion, the findings of this study provide foundational data for better understanding influences of fetal sex on brain development. Our study is expected to set the groundwork required to develop relevant pig models to better understand brain development.

## Figures and Tables

**Table 1:** Number of DEGs in developing male (M) and female (F) porcine fetal brains between GDs 45 vs. 60 and GDs 60 vs. 90. The direction of differential expression is also shown.

Comparison	Number of DEGs	Differential Expression
F.45 vs. F.60	801	Upregulated
F.45 vs. F.60	1258	Downregulated
F.60 vs. F.90	2665	Upregulated
F.60 vs. F.90	2660	Downregulated
M.45 vs. M.60	571	Upregulated
M.45 vs. M.60	752	Downregulated
M.60 vs. M.90	3163	Upregulated
M.60 vs. M.90	3442	Downregulated

**Table 2:** Number of significantly differentially expressed exons in the developing fetal brain of male and female fetuses between GDs 45 vs. 60 and GDs 60 vs. 90. The number in the parenthesis represents the number of genes associated with the exons.

	Female (GD45 vs. GD60)	Female (GD60 vs. GD90)	Male (GD45 vs. GD60)	Male (GD60 vs. GD90)
Downregulated	132 (99)	694 (491)	36 (29)	274 (215)
Upregulated	205 (148)	268 (200)	14 (12)	1772 (1222)
Total	337 (247)	962 (691)	50 (41)	2046 (1437)

**Table 3:** Pathways significantly over-represented by the DEGs of the developing fetal brain. The fetal sex and developmental periods (GDs) associated with each enriched pathway are shown. The FDR value shows the significance level of enrichment of the pathway.

Pathway	Number of DEGs	Fold Enrichment	FDR	Fetal Se×	Gestation Days
Ionotropic glutamate receptor pathway (P00037)	15	3.32	7.4 × 10^−3^	F	45 vs. 60
p53 pathway (P00059)	20	2.49	1.9 × 10^−2^	F	45 vs. 60
Endothelin signaling pathway (P00019)	19	2.47	2.3 × 10^−2^	F	45 vs. 60
Gonadotropin−releasing hormone receptor pathway (P06664)	48	2.3	1.3 × 10^−3^	F	45 vs. 60
Angiogenesis (P00005)	35	2.21	3.9 × 10^−3^	F	45 vs. 60
Wnt signaling pathway (P00057)	52	1.81	7.4 × 10^−3^	F	45 vs. 60
Gonadotropin−releasing hormone receptor pathway (P06664)	99	1.82	1.6 × 10^−4^	F	60 vs. 90
Angiogenesis (P00005)	68	1.65	4.0 × 10^−2^	F	60 vs. 90
Wnt signaling pathway (P00057)	30	1.99	3.4 × 10^−2^	M	45 vs. 60
Histamine H1 receptor mediated signaling pathway (P04385)	27	2.13	4.8 × 10^−2^	M	60 vs. 90
Endothelin signaling pathway (P00019)	48	1.96	1.1 × 10^−2^	M	60 vs. 90
Metabotropic glutamate receptor group III pathway (P00039)	39	1.93	4.0 × 10^−2^	M	60 vs. 90
Heterotrimeric G−protein signaling pathway−Gq alpha and Go alpha mediated pathway (P00027)	67	1.88	3.0 × 10^−3^	M	60 vs. 90
Angiogenesis (P00005)	88	1.75	3.2 × 10^−3^	M	60 vs. 90
Heterotrimeric G−protein signaling pathway−Gi alpha and Gs alpha mediated pathway (P00026)	81	1.69	6.5 × 10^−3^	M	60 vs. 90
EGF receptor signaling pathway (P00018)	65	1.6	5.0 × 10^−2^	M	60 vs. 90
CCKR signaling map (P06959)	76	1.53	4.5 × 10^−2^	M	60 vs. 90
Gonadotropin−releasing hormone receptor pathway (P06664)	102	1.53	1.2 × 10^−2^	M	60 vs. 90

## Data Availability

All the raw and processed data of this study have been submitted to the Gene Expression Omnibus database under the accession number GSE178970.

## Supplementary Materials

The following are available online at https://www.mdpi.com/article/10.3390/cells10092439/s1, Table S1: list of DE genes in male and female brains during GD45 to GD60 and GD60 to GD90. Table S2: list of genes showing significant alteration between male and female brains at same gestation day(s). Table S3: list of genes commonly altered during fetal brain development in pigs as well as humans. Table S4: list of genes showing significant alteration in the developing brain of male fetuses that are flanked by female fetuses compared to the brain of male fetuses flanked by a male on the medial side and a female on the lateral side. Table S5: exon-level expression changes in the male (M) and female (F) fetal brain during development from GD45 to GD60 and from GD60 to GD90. Table S6: number of exons that are either downregulated or upregulated between males (M) and females (F) during brain development (GD45 vs. GD60 and GD60 vs. GD90). Table S7: GO terms enriched among DEGs in the fetal brain. Table S8: comparative analysis of ON/OFF gene expression patterns between the fetal brain and placenta of pigs on GDs 60 and 90. Table S9: list of genes differentially expressed in the developing fetal brain that are known markers of brain cells.
